# Pain and Anxiety versus Sense of Family Support in Lung Cancer Patients

**DOI:** 10.1155/2014/312941

**Published:** 2014-07-13

**Authors:** Dimitra Lekka, Argiro Pachi, Athanasios Tselebis, Georgios Zafeiropoulos, Dionisios Bratis, Argiri Evmolpidi, Ioannis Ilias, Athanasios Karkanias, Georgios Moussas, Nikolaos Tzanakis, Konstantinos N. Syrigos

**Affiliations:** ^1^Psychiatric Department, “Sotiria” General Hospital of Chest Diseases, Mesogeion 152, 11527 Athens, Greece; ^2^Department of Endocrinology, “Elena Venizelou” Hospital, 11521 Athens, Greece; ^3^Department of Thoracic Medicine, Medical School, University of Crete, 71 003 Heraklion, Greece; ^4^Department of Social Medicine, Laboratory of Epidemiology, Medical School, University of Crete, 71 003 Heraklion, Greece; ^5^Oncology Unit, Third Department of Internal Medicine, Athens Medical School, “Sotiria” General Hospital of Chest Disease, Mesogeion 152, 11527 Athens, Greece

## Abstract

Lung cancer is a stressful condition for both patient and family. The anxiety and pain accompanying cancer and its treatment have a significant negative influence on the patient's quality of life. The aim of this study was to investigate the correlation between anxiety, pain, and perceived family support in a sample of lung cancer patients. The sample consisted of a total of 101 lung cancer outpatients receiving treatment at the oncology department of a general hospital. Anxiety, pain (severity and impact on everyday life), and perceived family support were assessed using Spielberger's State-Trait Anxiety Inventory, the Brief Pain Inventory, and the Family Support Scale, respectively. Statistical analyses revealed correlations between anxiety, pain, and family support as perceived by the patients. The intensity of pain had a positive correlation with both state and trait anxiety and a negative correlation with family support. Anxiety (state and trait) had a significant negative correlation with family support. In conclusion, high prevalence rates of anxiety disorders were observed in lung cancer patients. Females appeared more susceptible to anxiety symptoms with a less sense of family support. A negative correlation was evidenced between family support and anxiety and a positive one between anxiety and pain.

## 1. Background

Lung cancer is the most common cause of cancer-related death [1, 2]. Although mostly prevalent in male populations, owing to the increase of habitual smoking among female individuals, the prevalence of lung cancer among women is also rising [3–5]. Approximately 80–90% of patients suffering from lung cancer succumb to the ailment within the first year of diagnosis whilst only 15% live up to 5 years after initial diagnosis [1, 2].

Pain is one of the most common debilitating symptoms associated with lung cancer [6, 7] and is a serious issue not only for the patient in question but for the health personnel in general in terms of addressing the relevant symptoms [8]. Pain is defined as “an unpleasant sensory and emotional experience associated with actual or potential tissue damage” in addition to the subjective experience and account of the individual [9].

All lung cancer patients go through a pain assessment in order for the relevant symptoms to be better managed and hopefully ameliorated [6, 9].

Few data are available on the severity of the pain in different phases of cancer, and they were mostly inconclusive [10]. The same applied to the relation between the prevalence of cancer pain and the type of cancer, phase of disease, age, and gender [11]. Female cancer patients have been reported to experience more nociceptive pain when compared with male cancer patients, but meta-analyses and studies with more uniform populations of cancer patients have found no concrete evidence in support of such reported gender related symptom differences [12]. For people newly diagnosed with lung cancer no relationship was found between gender and pain, while controlling for age, comorbidities, and stage of cancer [13]. According to a population-based study in cancer patients, age and gender were not found to be significant predictors of the prevalence of pain, but stage of disease was a prominent predictor of moderate to severe pain [14]. Also terminal pain in lung cancer patients was independent of gender, age, performance status, stage, and histology [15].

Apart from the tortuous pain that they experience, cancer patients are confronted by a constant and vivid threat in addition to the associated intense anxiety related to the ailment [16, 17]. Anxiety is considered as an unpleasant emotional condition associated with a subjective sense of fear, bodily discomfort, and distress which could be related to a sense of an oncoming real or imagined threat [18].

In an effort to correlate possible demographic and disease-related variables to symptoms commonly associated with cancer, studies have found that younger patients and females are more likely to experience psychological distress [19] and/or symptoms of anxiety and depression [20]. Lung cancer patients appeared more anxious compared to individuals with other cancer diagnoses, and younger age, female gender, and advanced disease stage were associated with more anxiety symptoms [21]; yet the literature converges in suggesting that although there is a trend toward more distress in women and younger patients, disease stage is not predictive of emotional distress and histology type is not predictive of anxiety in lung cancer patients [22].

Lung cancer is a stressful condition not only for the patients in question but also for their family. The immediate family of a lung cancer patient has to deal with the significance of the cancer for the patient, the changes in everyday life, the numerous demands of caring for the patient which include observing and alleviating the relevant symptoms, monitoring, and maintaining the medication schedule as well as giving hope to the patient [23].

Sense of family support has been studied in other medical illnesses [24]. Research with diabetic patients indicate that sense of family support is positively associated with improvements in glycemic control and maintenance of glycosylated haemoglobin at acceptable levels [25, 26]. Greater levels of social support from spouses and other family members seem to contribute positively to the adaptation and prognosis of patients with end-stage renal disease and congestive heart failure [27–29]. Studies also report benefits from family support in bronchial asthma patients [30]. However, available literature on the role of family support in lung cancer patients, especially in relation with the subjective sensation of pain, is limited.

The aim of this study is to investigate whether pain among cancer patients is in any way related to anxiety and family support as perceived by the patient. In particular, this study will focus on the clinical characteristics of the sample population, attempting to identify whether or not there is a relation between pain and anxiety experienced by the patients, whether or not there is a relation between pain and perceived family support, and finally whether or not there is a relation between anxiety and perceived family support.

## 2. Methods

The study took place at the One Day Clinic of the Oncology Department of the Sotiria Hospital for Chest Diseases. The research was carried out from June to August 2013 and it was conducted on outpatients receiving daily chemotherapy (Daily Clinic). The Research and Ethic protocol was upheld.

### 2.1. Materials

The materials used in this study were: (a) Spielberger's State-Trait Anxiety Inventory (SSTAI), (b) the Brief Pain Inventory (BPI), and (c) the Family Support Scale (FSS). Demographic characteristics of patients (age, gender) and clinical variables (type and stage of cancer) were recorded.

#### 2.1.1. Spielberger's State-Trait Anxiety Inventory

Anxiety was assessed with the Spielberger State-Trait Anxiety Inventory (SSTAI) [31], one of the well-known and broadly used anxiety rating scales. The inventory consists of 40 items, each one graded from 1 to 4. The SSTAI differentiates anxiety to (a) anxiety caused by a specific condition (state subscale) and (b) anxiety as a more permanent characteristic of the personality (trait subscale). The SSTAI [31] is considered as having a high inner coherence reliability and validity compared to clinical diagnosis. Also it has been standardized and widely used in studies in the Greek population previously [32–35].

#### 2.1.2. Brief Pain Inventory (BPI)

The BPI consists of two parts. In the first part, items refer to the acuteness of perceived pain. In the second part, items refer to the extent to which the perceived pain has an impact on the patient's activities, mood, interpersonal relationships, sleep patterns, and quality of life [36].

#### 2.1.3. Family Support Scale (FSS)

To measure perception of family support we used, as in previous studies, FSS 13-item questionnaire. All 13 items of the scale (e.g., item 1: “My family supports me in all my efforts” and item 5: “I am always the one to blame when our home is a mess”) are rated on a five-point scale. The questionnaire's adaptation in modern Greek presented good reliability (alpha = 0.80). Individuals living by themselves did not answer the questionnaire [37, 38].

### 2.2. Statistical Analysis

Descriptive statistics were calculated for all study variables and data were expressed as mean ± standard deviation (SD) for continuous variables. The Kolmogorov-Smirnov test served as a goodness of fit test, utilized for normality analysis of the parameters and indicated the normality of the distribution. Independent samples *t*-test was used to reveal differences among continuous variables as to gender and type of cancer. ANOVA-Bonferroni was performed to identify differences among continuous variables as to disease stage. Pearson's *r* correlation coefficients were used to examine the univariate associations among continuous variables. Finally, to better determine the relationships among variables we performed stepwise multiple regression analysis. For regression models, an empirical approach was used after correlation analysis. Three models were investigated: (a) with pain severity as dependent variable and gender, age, trait and state anxiety, and family support as independent variables, (b) with pain interference total score as dependent variable and gender, age, trait and state anxiety, and family support as independent variables, (c) with state anxiety as dependent variable and gender, age, the two pain subscales, and family support scale as independent variables. All tests were two-sided and statistical significance was set at *P* < 0.05. All analyses were carried out using the statistical package SPSS version 19.

## 3. Results

Our sample consisted of 101 patients, 83 of which were male (82.2%) and 17 were female (17.8%, *x* ^2^ *P* < 0.01). The mean age of the female population was not statistically different than the corresponding of the male population, but females scored significantly higher in the subscale of pain severity (*P* < 0.05, Table 1) as well as in both anxiety subscales (*P* < 0.05 for trait anxiety and *P* < 0.01 for state anxiety, Table 1). On the contrary, the female population scored lower than males in the sense of family support scale (*P* < 0.01).

As to the type of cancer, 68 patients suffered from non-small cell lung cancer (NSCLC) and 33 from small cell lung cancer (SCLC). NSCLC patients were significantly younger than SCLC patients (64.10 ± 9.68 versus 68.36 ± 7.43, *P* < 0.05).

However, the type of cancer did not differentiate the scores in pain, anxiety, and family support scales (*P* > 0.05). As expected, NSCLC patients in stage IV scored higher in pain scales (ANOVA *P* < 0.05, Table 2), but scores in anxiety and sense of family support scales were irrespective of disease stage.

In state anxiety subscale 53% of male patients presented with anxiety symptoms (scored >45) compared to 73.3% of females who scored above 43. A total of 56.43% of patients (*N* = 57) presented with anxiety symptoms in state anxiety subscale.

In trait anxiety subscale 33.7% of male patients presented with anxiety symptoms (scored >41) compared to 61.1% of females who scored above 40. A total of 38.1% of patients (*N* = 39) presented with anxiety symptoms in trait anxiety subscale.

Observing the correlations between the scales, apart from the expected high correlations between the two anxiety subscales (*P* < 0.01, Table 3) and between the two pain subscales (*P* < 0.01Table 3), our results indicate high positive correlations between anxiety and pain scales. Instead, sense of family support was negatively correlated with both pain (*P* < 0.05, Table 3) and anxiety (*P* < 0.05, Table 3) scales. Correlations with age for any scale revealed no significant differences, possibly owing to the narrow width of age range.

To better determine the relationships among variables we performed stepwise multiple regression analysis, with pain severity as dependent variable and gender, age, trait and state anxiety, and family support as independent variables. Results revealed that state anxiety explained 10.8% of pain severity variance (*R* square change: 0.108, *F*: 9.92, *B*: 0.071, *P* < 0.01). Following the same procedure with pain interference total score as dependent variable and gender, age, trait and state anxiety, and family support as independent variables, only state anxiety predicted 16.1% of the variance of the dependent variable (*R* square change: 0.161, *F*: 15.72, *B*: 0.129, *P* < 0.01).

With state anxiety as dependent variable and gender, age, the two pain subscales, and family support scale as independent variables results indicate that family support scale explained 16.1% of anxiety variance (*R* square change: 0.161, *F*: 15.78, *B*: −0.82, *P* < 0.01) and pain interference was responsible for an additional 9.6% of anxiety variance.

## 4. Discussion

The high prevalence of anxiety disorders in cancer patients is well documented [39, 40]. There are many types of anxiety disorders, and all are relatively common in the population, with prevalence data varying from 10 to 15% for the Greek population [41, 42]. In general population one-month prevalence rates for all anxiety disorders range between 2.5 and 8.2%, 1-year rate is estimated around 17.2%, and lifetime prevalence is reported to be around 21–28.8% [41].

Anxiety prevalence rates in cancer patients remain high, even comparable to prevalence rates in other advanced diseases such as chronic obstructive pulmonary disease (COPD), where studies indicate a 37% trait anxiety rate for male COPD patients and a 46% rate for females [35]. According to the literature even higher rates of state anxiety could be regarded as untoward consequences of the treatment process that all patients undergo, exacerbated by the painful nature of the treatment procedures in combination with the uncertainty of the prognosis of the disease.

The female population of our sample appears to be particularly vulnerable to maladaptive anxious responses as evidenced by the high scores in relevant scales. Even though twice as many women have an anxiety disorder compared to men in the general population, women aged 45–54 scored the same as men on anxiety scales in the Greek population [43].

Higher levels of anxiety for the female population compared to men have been identified in other chronic diseases as well, such as bronchial asthma and chronic obstructive pulmonary disease [35].

Reduced sense of family support is a finding often revealed in studies in both healthy Greek population [37, 38] and in groups of Greek patients [26, 30] and could be explained by the traditional definition of the female gender role and responsibilities in a prevalent social context and in a particular family structure.

Type and stage of cancer do not differentiate anxiety levels. It is possible that patients construe disease seriousness subjectively, which contributes to the development of psychological distress symptoms. Another explanation to justify the absence of correlation between illness severity and anxiety symptoms could possibly be associated with the role of family support. In other words, a less favorable prognosis announced to the family environment has the ability to moderate or minimize relevant requirements from the part of the patient, while increasing benefits and support to the seriously affected member. The increase in the sense of family support contributes to the reduction of negative factors acting favorably in order to mitigate the psychological burden that is expected to be brought by the severity of the disease [44]. However this theory necessitates further investigation as it requires that particular family values and patterns exist like those still found in Greece and in the Mediterranean countries.

In a recent review of the literature, the prevalence of pain in all cancer types was more than 50% [45]. The high prevalence of anxiety disorders in those with chronic pain is also well documented, with prevalence data of 20–40% [46]. Pain and stress circuits are strongly correlated with one another communicating through overlapping neuronal pathways [47] and sharing common neurotransmitter regulatory systems, mainly serotonin and norepinephrine [48].

Anxious patients may experience higher levels of pain through the activation of distress pathways [49]. This activation in limbic and paralimbic regions during anxiety states may disrupt the descending inhibitory pain pathways [50], since, for example, frontal-amygdalar circuits may modulate the affective intensity of pain [51, 52].

Although the method and the purpose of the study are not aimed at recording causal relationships, it is likely that the reduced sense of family support results in increased anxiety symptoms, which in turn increases the pain sensation.

An alternative explanation would suggest that patients with a greater vulnerability to pain are more susceptible to anxiety symptoms [53], which in turn affect the subjective sense of the received family support.

It should be emphasized that both theories represent a linear context approach, which remains within the limits of doubt, unless it can be scientifically proven. A circular causation theory could equally serve as possible explanation, indicating that the unfavorable change in one of the factors would lead to successive changes in others which in turn will negatively affect the original [54, 55].

However, altering the sense of family support is the only nonpharmaceutical therapeutic intervention and will therefore be advisable for the future studies to determine and verify whether improvements with certain psychotherapeutic techniques will effectuate significant clinical changes in anxiety and pain for the lung cancer patient.

## 5. Conclusions

High prevalence rates of anxiety disorders are observed in lung cancer patients. Females appear more susceptible to anxiety symptoms with a less sense of family support. A negative correlation is evidenced between family support and anxiety and a positive one between anxiety and pain.

## Limitations of the Current Study

The current study is limited by the sample's size and characteristics. The sample size limits the generalization of the study's findings. The sample consisted exclusively of outpatients.

## Figures and Tables

**Table 1:** Means (SD) in pain and anxiety scales as to gender.

Gender	Age	Pain severity score	Pain interference score	State anxiety score	Trait anxiety score	Family support score
Male						
Mean	65,73	2,72	3,91	46,14	37,44	59,91
*N*	83	83	83	83	83	70
SD	8,92	1,99	3,10	9,13	7,84	4,32

Female						
Mean	64,39	3,90	5,27	53,89	42,44	54,07
*N*	18	18	18	18	18	14
SD	10,57	2,48	2,92	10,20	10,09	3,27

Total						
Mean	65,49	2,93	4,15	47,52	38,33	58,94
*N*	101	101	101	101	101	84
SD	9,19	2,12	3,10	9,74	8,45	4,69

**Table 2:** Pain scores as to NSCLC stage.

	Pain severity score	Pain interference score
II		
Mean	,8125	,1786
*N*	4	4
SD	1,62500	,35714

III		
Mean	1,7692	2,1758
*N*	13	13
SD	1,90521	2,43148

IV		
Mean	3,1716	4,8375
*N*	51	51
SD	1,91670	2,92924

Total		
Mean	2,7647	4,0546
*N*	68	68
SD	2,01389	3,08485

**Table 3:** Correlations between pain and anxiety scales.

	Age	Pain severity score	Pain interference score	State anxiety score	Trait anxiety score
Pain severity score					
Pearson correlation	,034				
Sig. (2-tailed)	,737				
*N*	101				

Pain interference score					
Pearson correlation	,060	,843			
Sig. (2-tailed)	,554	,000			
*N*	101	101			

State anxiety score					
Pearson correlation	,022	,395	,446		
Sig. (2-tailed)	,829	,000	,000		
*N*	101	101	101		

Trait anxiety score					
Pearson correlation	−,039	,312	,363	,665	
Sig. (2-tailed)	,700	,001	,000	,000	
*N*	101	101	101	101	

Family support score					
Pearson correlation	,043	−,309	−,251	−,402	−,536
Sig. (2-tailed)	,696	,004	,022	,000	,000
*N*	84	84	84	84	84

Correlation is significant at the 0.01 level (2-tailed).

Correlation is significant at the 0.05 level (2-tailed).
